# Platelet and Red Blood Cell Counts, as well as the Concentrations of Uric Acid, but Not Homocysteinaemia or Oxidative Stress, Contribute Mostly to Platelet Reactivity in Older Adults

**DOI:** 10.1155/2019/9467562

**Published:** 2019-01-16

**Authors:** Kamil Karolczak, Bartlomiej Soltysik, Tomasz Kostka, Piotr J. Witas, Cezary Watala

**Affiliations:** ^1^Chair of Biomedical Sciences, Department of Haemostatic Disorders, Faculty of Health Sciences, Medical University of Lodz, ul. Mazowiecka 6/8, 92-215 Lodz, Poland; ^2^Clinic of Geriatrics, Faculty of Health Sciences, Medical University of Lodz, ul. Pieniny 30, 92-115 Lodz, Poland

## Abstract

**Purpose:**

The goal of this study was to estimate the hierarchical contribution of the most commonly recognized cardiovascular risk factors associated with atherogenesis to activation and reactivity of blood platelets in a group of men and women at ages 60-65.

**Methods:**

Socioeconomic and anthropometric data were taken from questionnaires. Blood morphology and biochemistry were measured with standard diagnostic methods. Plasma serum homocysteine was measured by immunochemical method. Plasma concentrations of VCAM, ICAM, total antioxidant status, and total oxidant status were estimated with commercial ELISA kits. Markers of oxidative stress of plasma and platelet proteins (concentrations of protein free thiol and amino groups) and lipids (concentrations of lipid peroxides) and generation of superoxide anion by platelets were measured with colorimetric methods. Platelet reactivity was estimated by impedance aggregometry with arachidonate, collagen, and ADP as agonists. Expression of selectin-P and GPIIb/IIIa on blood platelets was tested by flow cytometry.

**Results:**

Platelet aggregation associated significantly negatively with HGB and age and significantly positively with PLT, MPV, PCT, PDW, and P-LCR. When platelet reactivity (“cumulative platelet reactivity_aggregation”) was analyzed in a cumulated manner, the negative association with serum concentration of uric acid (*R*_s_ = −0.169, *p* = 0.003) was confirmed. Multivariate analysis revealed that amongst blood morphological parameters, platelet count, plateletcrit, and number of large platelets and uric acid are the most predictive variables for platelet reactivity.

**Conclusions:**

The most significant contributors to platelet reactivity in older subjects are platelet morphology, plasma uricaemia, and erythrocyte morphology.

## 1. Introduction

Ageing is associated with a plethora of changes in the physiology of the haemostatic system. This statement appears as trivial as it is true. However, it is not entirely clear which qualitative and quantitative changes specifically occur in some particular “compartments” of haemostatic system, like for instance blood platelets. Since the risk of atherosclerosis increases with age, it seems obvious that the platelet-derived prothrombotic state also associates with age, but such a statement has not been unambiguously reported [1].

Major quantitative and qualitative changes in blood platelets associated with ageing may be roughly attributed to one of two groups of factors: those associated with blood morphological parameters and those concerning a biochemical “status” of blood plasma [1].

Morphological changes that aggravate with age, which may be important in shaping the procoagulant phenotype of platelets, probably predominantly concern blood platelet count and volume [1]. These two morphological features of blood platelets have been nonoccasionally reported to associate with higher thrombotic risk [2–4]. It should be noted herein that platelets are capable to interact with all types of cells circulating in peripheral blood [5–12]. Thus, the morphological changes of erythrocytes and white blood cells, especially monocytes and neutrophils, may have a significant impact on atherosclerosis-associated platelet activation and reactivity [13]. Thus, it is highly plausible that tracking of blood platelets and other blood cells capable to interact with platelets may at least serve as screening method utilized for a preliminary estimation of thrombotic risk in the elderly.

The biochemical changes taking place in the external environment, in which blood platelets function, concern mainly the fluctuations in concentration of pro- and antiatherogenic molecules, which are attributed—in an unquestionable manner—to the pathogenesis of platelet-driven atherosclerosis. The list of these molecules includes mainly LDL cholesterol, HDL cholesterol [14–17], and glucose [18–20], but we also have to be aware that molecules with a more controversial role in atherogenesis may matter, like uric acid [21–23] or homocysteine [24].

A significant role in the pathogenesis of atherosclerosis is attributed to oxidative stress [25]. The shift of redox equilibrium in favor of the oxidative reactions, perpetuated by the overproduction of reactive oxygen species, leads to peroxidation of biomacromolecules, with a more specific reference to lipid peroxidation or oxidation of thiol and amino groups in platelet and plasma proteins. These processes have an undeniable activating effect on platelets *in vitro* [26, 27]. Their effects on platelets *in vivo* are more disputable, which is mainly due to the fact that some antioxidant therapies have not been proven to significantly modulate platelet pathophysiological activation in ageing-associated atherogenesis [28].

A brief overview of the most important groups of factors involved in the atherosclerotic process shows the overall complexity of biochemical stimuli to which blood platelets may respond. For simplicity, these stimuli have been usually tested individually and separately. In this study, we made an attempt to simultaneously assess the contribution of each of the above groups of factors to shape platelet atherogenic potential. To comprehensively analyze the particular impacts of potential risk factors in the shaping of blood platelet activation and reactivity, we have focused our attention on the following parameters: blood cell morphology, changes in serum pro- and antiatherosclerotic factors, as well as the oxidative stress measured as the degree of oxidative damage of proteins and lipids in blood plasma and platelet proteins and the capacity of blood platelets to generate superoxide anion. We have statistically evaluated the potential of the above factors to influence the ability of blood platelets to aggregate after *in vitro* stimulation with arachidonic acid (AA) or collagen (COL) and to expose some platelet surface membrane glycoproteins, like P-selectin and the active form of fibrinogen receptor (GPIIb/IIIa glycoprotein) in both circulating resting blood platelets and after *in vitro* platelet agonizing with arachidonic acid, collagen, or ADP.

The specific goal of this study was to estimate the hierarchical contribution of the most commonly recognized cardiovascular risk factors associated with atherogenesis to the functional state of blood platelets, i.e., their activation and reactivity. The suspected factors were analyzed individually as well as in three subsets of larger clusters of factors, referred to as morphological, biochemical, and oxidative factors. These investigations were undertaken in the group of elderly people of both sexes—the individuals suspected to exhibit the higher risk of multifactorial haemostatic risk.

## 2. Materials and Methods

### 2.1. Chemicals

Phosphate-buffered saline (PBS) was from Avantor Performance Materials Poland S.A. (Gliwice, Poland). Arachidonate, collagen, and ADP were from Chrono-Log Corp. (Havertown, PA, USA). Dimethyl sulfoxide, cytochrome c, sodium dodecyl sulphate (SDS), Ellman's reagent (5,5′-dithiobis-2-nitrobenzoic acid, DTNB), glutathione (reduced), HCl, 2,4,6-trinitrobenzenesulphonic acid (TNBS), ethanol, ethyl acetate, guanidine hydrochloride, xylenol orange, Fe(NH_4_)_2_(SO_4_)_2_, and perchloric acid were from Sigma-Aldrich (St. Louis, MO, USA). Pierce™ BCA Protein Assay Kit was from Thermo Fisher Scientific (Waltham, Massachusetts, USA). Fluorolabelled monoclonal antibodies (moAbs)—anti-CD61/PerCP, antiCD62/PE, PAC-1/FITC, isotype antibodies, and CellFix were from Becton Dickinson (San Diego, CA, USA).

### 2.2. Design of the Study and Subject Recruitment

Our present study presents the results obtained in subgroups of volunteers participating in the project entitled “The occurrence of oxidative stress and selected risk factors for cardiovascular risk and functional efficiency of older people in the context of workload” (funded by the Central Institute For Labour Protection-National Research Institute, Warsaw, Poland, and supervised by the Clinic of Geriatrics at Medical University in Lodz, Poland). Two basic inclusion criteria were ages within the range of 60 to 65 years and the willingness to participate [29]. To ensure approximately equal participation of men and women, a stratified probability sampling algorithm was used. The research group included roughly 350 subjects (approx. equal sex proportions of 175 men and 175 women), aged from 60 to 65 years, who responded to the invitations (a source population). According to the outcomes of our preliminary pilot study, minimal correlation between the cumulative measure of platelet aggregation and the leading variables describing blood platelet morphology (number of blood platelets (PLT), plateletcrit (PCT), and platelet large cell ratio (P-LCR)) was assumed to be at least 0.22-0.23; the statistical test power, i.e., the likelihood of rejecting false null hypothesis, was assumed to be 90%, and the significance level was assumed to be at least 1% [30]. Of the enlisted target population of sex-equilibrated group of about 300 individuals, we randomly selected 125 women and 126 men (simple unrestricted randomization) to create the subgroup for studying platelet reactivity and blood platelet morphology parameters.

All steps of experiments with the participation of human subjects were undertaken under the guidelines of the Helsinki Declaration for human research. The study was approved by the Committee on the Ethics of Research in Human Experimentation at Medical University of Lodz. Written abstract of experiment, including detailed information regarding the study objectives, study design, risks, and benefits, was given to each of volunteers during recruitment procedure to give an opportunity to consider all pros and cons in regard of participation in the study. At the beginning of the study, the respondents obtained information about purpose, the course and the use of possible results of the research, as well as the opportunity to refuse to participate in the study at any stage of the experiment, without providing the reason.

The inclusion criterion in the study was the ages between 60 and 65 years and an agreement to participate in the study (confirmation in the written consent form), well-defined clinical status, and stable pharmacotherapy (drugs not changed for at least 3 months). We excluded subjects with acute coronary syndromes or any acute medical events within 6 months of randomization, patients suffering for psychiatric diseases, alcohol abusers, or those consuming alcohol the day before blood withdraw, patients being in the course of chemo- or radiotherapy, and subjects with the signs of acute inflammation or with infections, taking antiplatelet drugs (acetylsalicylic acid, clopidogrel) or analgesics within 14 days before blood sampling. Forty-nine subjects were disqualified due to the use of antiplatelet drugs (acetylsalicylic acid, clopidogrel). Sociodemographic, medical anamnesis, and anthropometric data were obtained, and medical examinations were performed in the Department of Geriatrics at Medical University (Lodz, Poland).

The tested group consisted of 251 respondents, 49.4% were women. The median of age was 63 (IQR: 61-64 years, 33.9% worked intellectually and 34.3% manually, and 31.9% subjects were unemployed). The median education was 13 years and 22.7% were active smokers. Amongst 251 participants, 46.6% were diagnosed with arterial hypertension, 62.5% had hypercholesterolemia, 9.2% suffered from type 2 diabetes mellitus, 1.6% of individuals had myocardial infarction, while 2.8% had stroke in the past. The group of 12.4% of the subjects reported chronic obstructive pulmonary disease, 48% had osteoarthritis, and 11.2% had osteoporosis.

Median body mass index was 27.4 (IQR: 24.8-30.3), and 32.3% had BMI exceeding 30 kg/m^2^ (obesity). The proportion of 20.7% of the subjects had taken beta-blockers, 10% had calcium channel blockers, 15.5% had diuretics, and 24.7% took other antihypertensive agents. The proportion of 17.5% of the subjects took statins and 8.8% used antidiabetic drugs.

The characteristics of blood morphology, serum biochemistry, and blood platelet reactivity parameters in the studied subjects are given in Table 1.

### 2.3. Basic Laboratory Measurements: Haematology and Serum Biochemistry

Parameters of blood morphology were measured with haematological analyzer 5-Diff Sysmex XS-1000i (Sysmex, Kobe, Japan). Biochemical parameters in serum/plasma were estimated with the analyzer DIRUI CS 400 (Dirui, Changchun, China). The concentration of homocysteine was measured using the analyzer Immulite 2000 XPi (Siemens, Erlargen, Germany).

### 2.4. Platelet Aggregometry

Platelet aggregability was measured with multiplate analyzer—impedance aggregometer (Dynabyte, Munich, Germany)—according to the protocols used earlier [31, 32], with arachidonate, collagen, and ADP as the agonists of blood platelets. In brief, samples of whole blood were recovered for 10 minutes at room temperature. The aliquots of a whole blood (300 *μ*l) were added to multiplate analyzer cells filled with 0.9% NaCl (300 *μ*l) (preheated at 37°C) and mixed for 3 minutes. After this time, platelet agonists, arachidonate, collagen, and ADP at the final concentrations of 0.5 mmol/l, collagen (1 *μ*g/ml), or ADP (10 *μ*mol/l), respectively, were added to the measurement cells to trigger platelet aggregation. The stock solutions of platelet agonists were prepared according to the manufacturer's protocols. The following parameters of platelet aggregometry were recorded: maximal platelet aggregation (*A*_max_), area under the aggregometric curve (AUC), and the integrative indicator of platelet reactivity, estimated according to the following formula: (*A*_max_ × AUC)/1000.

### 2.5. Isolation of Blood Platelets

Blood platelets were isolated according to the procedure described in details earlier [33]. Briefly, whole blood was centrifuged at 190 ×*g* (12 min, 37°C) and top layer (platelet-rich plasma) was harvested and further centrifuged at 2000 ×*g*/12 min/37°C. The resulting pellet of thus separated blood platelets was suspended in a Tyrode's buffer (10 mmol/l HEPES, 140 mmol/l NaCl, 3 mmol/l KCl, 0.5 mmol/l MgCl_2_, 5 mmol/l NaHCO_3_, and 10 mmol/l C_6_H_12_O_6_, pH 7.4) and final platelet count was adjusted to 1 × 10^8^ platelets/ml. Platelet titer was estimated by a spectrophotometric method [34].

### 2.6. Flow Cytometry of Blood Platelets

Flow cytometric measurements were undertaken in order to measure of the activation of circulating platelets and platelet reactivity in response to agonists after *in vitro* stimulation with 0.5 mmol/l arachidonate or 20 *μ*g/ml collagen included the determinations of the expressions of the activated glycoprotein complex GPIIb/IIIa and P-selectin. Flow cytometric analyses were done according to the protocol published recently elsewhere [35]. Briefly, samples of a whole blood were activated for 5 minutes at room temperature with the mentioned agonists. After activations, blood samples were stained with the gating antibody anti-CD61 and either anti-CD62P (P-selectin) or PAC-1 (anti-GPIIb/IIIa) antibodies (stained with PerCP, phycoerythrin, and fluorescein isothiocyanate, respectively) for 20 minutes at room temperature. Relevant isotype IgG antibodies were used as a negative control. During the staining procedure, the samples were protected from light. After staining, 300 *μ*l of PBS was added to each sample and 10000 events (CD61/PerCP-positive objects) were analyzed in each sample with a FACSCanto II flow cytometer (Becton Dickinson, Franklin Lakes, NJ, USA).

### 2.7. Estimation of the Markers of Oxidative Stress in Blood Platelets and Plasma

Concentrations of free sulfhydryl groups in platelet and plasma proteins were estimated according to the methods reported earlier by Ando and Steiner [36]. Briefly, to the samples of plasma and the solutions of platelet proteins, 10% SDS and 10 mmol/l phosphate buffer (pH 8.0) were added, and after that, the absorbance at *λ* = 412 nm was measured (*A*_0_). Next, the solution of 5,5′-dithiobis(2-nitrobenzoic) acid (DTNB), prepared in 10 mmol/l phosphate buffer (0.4 mg DTNB/ml of phosphate buffer), was added and the absorbance at *λ* = 412 nm was read again (*A*_1_). The calculated differences of *A*_1_-*A*_0_ served to estimate the concentrations of free amino groups, using the millimolar extinction coefficient of *ε* = 13.6 mmol^−1^ × l × cm^−1^.

The concentrations of free amino groups in platelet and plasma proteins were estimated according to Sashidhar et al. [37]. In brief, the samples of blood plasma and lysates of blood platelets were mixed with 2,4,6-trinitrobenzenesulfonic acid (TNBS, 0.01% solution prepared in 0.1 mol/l sodium bicarbonate buffer, pH 8.5) and left in darkness for 2 h at 37°C. Next, SDS (10%) and HCl (1 mol/l) were added to the samples. The concentration of the colored product was estimated on the basis of the absorbance measured at *λ* = 335 nm.

For the measurement of lipid hydroperoxides, we used spectrophotometric method described by Bartosz [38]. Blood plasma and platelet lysates were mixed with the solution of Fe(NH_4_)_2_(SO_4_)_2_ prepared in 1.1 mol/l perchloric acid and left for 30 min at room temperature. The absorbance of the colored product was measured at *λ* = 560 nm. The concentration of lipid hydroperoxides was estimated on the basis of the standard curve prepared for *t*-butyl hydroperoxide.

Total antioxidant status (TAS) and total oxidant status (TOS) in collected samples of blood plasma were evaluated with commercially available kits from LDN Labor Diagnostika Nord GmbH & Co. KG (Nordhorn, Germany). Concentration of plasma protein and the protein content in platelet lysates was estimated with the Pierce™ BCA Protein Assay Kit.

### 2.8. Measurements of the Markers of Endothelial Dysfunction

Plasma concentrations of von Willebrand factor, soluble VCAM and soluble ICAM, were measured with commercially available ELISA kits provided by Abcam (Cambridge, UK).

### 2.9. Measurements of Superoxide Anion Generation by Blood Platelets

Spectrophotometric method of cytochrome c reduction by superoxide anion, described by Gresele et al. [39], was used to determine the concentrations of generated superoxide radical. The suspension of isolated blood platelets in Tyrode's buffer, adjusted to 1 × 10^8^ platelets/ml, was mixed with the solution of bovine cytochrome c prepared in PBS. The final concentration of cytochrome c was 80 *μ*mol/l. Next, PBS or homocysteine (Hcy) (final concentration 25 *μ*mol/l) was added to blood platelets in cytochrome c solution and incubated at room temperature for 15 minutes. Changes in the absorbance of cytochrome c were measured spectrophotometrically at *λ* = 550 nm, and the concentration of superoxide anion was estimated on the basis of the molar extinction coefficient of *ε* = 18700 mol^−1^ × l × cm^−1^.

### 2.10. Statistical Analysis

Data were expressed as mean + SD or median and IQR (interquartile range: lower (25%) to upper quartile (75%)), depending on data distribution and variance homogeneity (Shapiro-Wilk's test and Brown-Forsythe's test, respectively) and the scale of data (continuous vs. categorical). We analyzed the possible outliers with the use of either Grubb's or Tukey's tests. The missing data (if occurring less often than 3% per a variable) were imputed using the *k* nearest neighbor analysis. Student's *t*-test for independent samples or Mann-Whitney *U* test was used to compare the groups, depending on whether the data met the assumptions of data normality and homoscedasticity. The comparisons with the adjustments for dummy or confounding variables were performed using an analysis of covariance (ANCOVA). Simple correlations were estimated with the LOO-boosted rank Spearman correlation test, while partial and semipartial correlations were evaluated with the use of multiple regression analysis on raw or Box-Cox-transformed data. The correlation and regression models were validated using the leave-one-out (LOO, jackknife or jackknife-d) techniques and bootstrap-boosted procedures (1000-10000 iterations), respectively. Multiple group comparisons were performed with various models of the analysis of variance, followed by multiple comparisons post hoc tests (Tukey's honest significant difference test, Fisher's least significant difference test, or Dunnett's test). To rank the predictors of the best discrimination between the groups ascribed as to either the lower or the higher blood platelet reactivity, we employed a panel of classical statistical methods and data mining classifiers, including multiple logistic regression, linear discriminant analysis, canonical analysis, MAR Splines regression, support vector machine method, naïve Bayes classifier, and *k* nearest neighbors (*kNN*) method. The outcomes of these analyses were further used to select the best predictors. The goodness of fit for the logistic regression models was evaluated using the Hosmer-Lemeshow test [40]. The variables describing platelet aggregability in response to agonists and platelet surface membrane glycoprotein expressions were subjected to normalization according to the van der Waerden method. Cumulative normal scores through the used agonists were determined for the marker of whole blood aggregometry (*A*_max_) and two flow cytometry markers (CD62P and PAC-1), and such cumulative measures were further dichotomized according to the median in such a way that the values below or equal to median (≤Me, rank 0) corresponded to the “lower blood platelet reactivity”, while the values above median (>Me, rank 1) corresponded to the “higher blood platelet reactivity”. Such categorical measures of platelet reactivity were accepted as the grouping variables or dichotomous dependent variables in some multivariate analyses. Other variables selected as the best predictors were dichotomized according to the median using the same calculus algorithm and the outcomes employed to construct Cochrane plots. In the statistical analyses, we used the following software: the Statistica v. 13.1 (Statsoft), StatsDirect v.3.0.182, Resampling Stats Add-in for Excel v.4, and GraphPad Prism v.5.

## 3. Results

### 3.1. Comparisons between Individuals with Lower and Higher Blood Platelet Reactivity

To discriminate the individuals with lower or higher blood platelet reactivity, we used the dichotomized values of the van der Waerden normal scores of *A*_max_ cumulated through the agonists used (AA, collagen, and ADP), referred to as the “cumulative reactivity_aggregation,” or the dichotomized values of the van der Waerden normal scores of the so-called “cumulative global platelet reactivity,” cumulated through the methods (aggregometry and flow cytometry), the surface platelet membrane antigens (P-selectin, the active GPIIb/IIIa complex), and the agonists used (AA, collagen, and ADP). *Per analogiam*, we estimated the “cumulative activation_flow cytometry” (through P-selectin and the active GPIIb/IIIa in circulating/resting platelets), the “cumulative P-selectin expression”, or the “cumulative active GPIIb/IIIa expression” (through agonists: AA and collagen).  Table 2 presents the selected blood morphology and plasma/serum biochemical parameters determined in the individuals showing either lower (group 1) or higher platelet reactivity (group 2) (“cumulative global platelet reactivity”). Subjects with higher indices of platelet reactivity exhibited higher values of all parameters of platelet morphology as well as the increased number of white blood cells and slightly, but significantly, higher number of neutrophils. Uric acid was a little higher in those with lower platelet reactivity, although the difference was at the border of statistical significance.

### 3.2. Simple Associations between Blood Platelet Reactivity and Blood Morphology or Biochemical Parameters

The evaluation of the correlations between the markers of platelet reactivity and the chosen morphological and biochemical blood parameters, not adjusted for any confounding variables except of sex, was performed for nontransformed raw variables depicting platelet reactivity and for variables cumulated through the used agonists. Such a “fusion” of aggregometric data obtained for all three agonists enabled us to describe a comprehensive reactivity of blood platelets (“cumulative platelet reactivity_aggregation”).

PLT (number of blood platelets) and plateletcrit (PCT) were positively associated with platelet responsiveness to all used agonists (*R*_s_ = 0.173, *p* = 0.027; *R*_s_ = 0.283, *p*<<0.0001; and *R*_s_ = 0.345, *p*<<0.0001 for correlations between PLT and AA-, COL-, and ADP-dependent reactivity, respectively, and *R*_s_ = 0.182, *p* = 0.0014; *R*_s_ = 0.312, *p*<<0.0001; and *R*_s_ = 0.423, *p*<<0.0001 for correlations between PCT and AA-, COL-, and ADP-dependent reactivity, respectively). P-LCR (platelet large cell ratio) positively associated with AA- and ADP-induced platelet reactivity (*R*_s_ = 0.123, *p* = 0.032 for AA and *R*_s_ = 0.176, *p* = 0.0021 for ADP). COL-dependent aggregation appeared as not shaped by P-LCR (data not shown). MPV (mean platelet volume) (*R*_s_ = 0.169; *p* = 0.003) and PDW (platelet distribution width) (*R*_s_ = 0.173, *p* = 0.002) were significantly associated only with the aggregability of platelets stimulated by ADP.

Number of monocytes appeared as associated with platelet responsiveness to AA (*R*_s_ = −0.121, *p* = 0.035), whereas number of neutrophils was found to correlate with platelet responsiveness to COL (*R*_s_ = 0.121, *p* = 0.035) and to ADP (*R*_s_ = 0.120, *p* = 0.037).

Amongst variables describing red blood cells, RBC (count of red blood cells), HGB (concentration of hamoeglobin), and HCT (haematocrit) were negatively significantly associated with platelet aggregation, but only when triggered by ADP (*R*_s_ = −0.127, *p* = 0.028; *R*_s_ = −0.206, *p* = 0.0003; and *R*_s_ = −0.152, *p* = 0.008).

Age was found as a factor negatively associated with platelet aggregability induced by AA (*R*_s_ = −0.113, *p* = 0.049) and ADP (*R*_s_ = −0.118, *p* = 0.04), but not with COL (data not shown).

For cumulative platelet reactivity_aggregation data, we have found that HGB (*R*_s_ = −0.125, *p* = 0.031), PLT (*R*_s_ = 0.313, *p*<<0.0001), MPV (*R*_s_ = 0.145, *p* = 0.012), PCT (*R*_s_ = 0.359, *p*<<0.0001), PDW (*R*_s_ = 0.150, *p* = 0.009), P-LCR (*R*_s_ = 0.158, *p* = 0.062), and age (*R*_s_ = −0.138, *p* = 0.016) remain significant associates.

The analysis of correlations between chosen biochemical serum markers and platelet reactivity to individual agonists revealed the following associations.

Both AA- and ADP-dependent platelet aggregations were significantly associated in a negative manner with the concentrations of uric acid (*R*_s_ = −0.140; *p* = 0.015 and *R*_s_ = −0.184, *p* = 0.001 for correlations between uric acid and AA- and ADP-dependent platelet aggregation, respectively). On the other hand, platelet reactivity to AA and ADP remained positively associated with the concentrations of lipid peroxides in blood platelets (*R*_s_ = 0.147; *p* = 0.01 and *R*_s_ = 0.149, *p* = 0.009, for associations noted between concentration of platelet lipid peroxides and platelet aggregability induced with AA and ADP, respectively). Moreover, AA-triggered platelet aggregation was found to be significantly associated with the concentrations of free thiol groups in blood platelets (*R*_s_ = 0.116, *p* = 0.043). In turn, COL-dependent platelet aggregation was found to be significantly associated only with concentration of free thiol groups in blood plasma (*R*_s_ = 0.116, *p* = 0.045).

When platelet reactivity (“cumulative platelet reactivity_aggregation”) was analyzed in a cumulated manner, the negative association with serum concentration of uric acid (*R*_s_ = −0.169, *p* = 0.003) was confirmed.

### 3.3. Predictors of Lower and Higher Reactivity of Blood Platelets: Multivariate Analyses

The purpose of this part of analysis was to check out which sets of variables and which variables in these sets predict with the greatest accuracy the higher and lower reactivity of blood platelets. The variables were grouped into the following clusters of variables: (1) morphological variables (parameters of morphology of erythrocytes, leukocytes, and platelets), (2) metabolic variables, including lipidogram (total cholesterol and cholesterols of lipoprotein fractions LDL and HDL) and other markers, like fasting glycaemia, uric acid, homocysteine, thromboxane, VCAM, ICAM-1, and von Willebrand factor, and (3) the markers of oxidative stress of blood plasma and blood platelets. In order to determine which explanatory variables are the most significant predictors discriminating between subjects with lower and higher reactivity of blood platelets, we used a panel of multivariate data mining analysis (MAR Splines regression, support vector machine method, naïve Bayes classifier, and *k* nearest neighbors (*kNN*)). The correctness of the outcomes of all the abovementioned methods with a true (real) allocation to either group (lower or higher platelet reactivity) was evaluated with the use of the algorithm known as the voting of *k* judges. The *kNN* (particularly with the use Czebyszew's distance, 90.2% correct) and support vector machine method (89.3% correct) appeared as the most correct (the most compatible with a real allocation). The compatibility with two other methods of analysis, MAR Splines regression and naïve Bayes classifier, did not exceed 75% of correctness.

Using this algorithm, we estimated that for the model of platelet reactivity, the most predictive variables amongst blood morphological parameters were platelet count (*χ*^2^ = 27.9, *p* < 0.0005), PCT (*χ*^2^ = 32.7, *p* < 0.0002), and number of large platelets (*χ*^2^ = 22.2, *p* < 0.01). Amongst metabolic variables uric acid (*χ*^2^ = 21.1, *p* < 0.02), free amino groups in platelet proteins (*χ*^2^ = 17.4, *p* < 0.05) and vWF (*χ*^2^ = 16.6, *p* = 0.055) appeared the best predictors, while amongst free radical parameters, the most predictive variables were TAS (*χ*^2^ = 17.4, *p* < 0.05) and superoxide radical generation in platelets in the absence of Hcy (*χ*^2^ = 10.4, *p* = 0.104). For the model of platelet activation, the most discriminative were free amino groups in platelet proteins (*χ*^2^ = 19.2, *p* < 0.005), uric acid (*χ*^2^ = 20.9, *p* < 0.02), superoxide radical generation in platelets in the absence or presence of Hcy (resp., *χ*^2^ = 17.6, *p* < 0.01 and *χ*^2^ = 15.1, *p* < 0.05), and TOS (*χ*^2^ = 12.4, *p* < 0.05).

We performed a multiple logistic regression analysis to answer the question of how selected analyzed (confounding/coexplanatory) variables contribute to lower or higher blood platelet reactivity cumulated through the used agonists (AA, collagen, and ADP). The dependent variables were the dichotomized values of the variables referred to as “cumulative platelet reactivity aggregation” or “cumulative global platelet reactivity” (see legend to Table 2). When adjusted for age and gender, the following variables appeared individually as the significant predictors of blood platelet reactivity: leukocyte count (OR = 5.46 (95% CI: 1.40-20.31), *p* < 0.015), platelet count (OR = 1.27 (95% CI: 1.11-1.45), *p* < 0.001), PCT (OR = 1.01 × 10^7^ (95% CI: 3.16 × 10^3^-3.21 × 10^10^), *p*<<0.0001), P-LCR (OR = 1.03 (95% CI: 1.00-1.07), *p* < 0.05), uric acid (OR = 0.58 (95% CI: 0.44-0.95), *p* < 0.05), free amino groups in platelet proteins (OR = 4.96 (95% CI: 1.04-20.21), *p* < 0.05), and free SH groups in platelet proteins (OR = 1.16 (95% CI: 1.01-1.34), *p* < 0.04).

Upon the overall multiple post hoc standardization for platelet and leukocyte counts, PCT, P-LCR, uric acid, free SH groups, and free amino groups in platelet proteins, merely PCT (OR = 2.70 × 10^4^ (95% CI: 2.18–3.36 × 10^8^), *p* < 0.04), P-LCR (OR = 1.04 (95% CI: 1.00-1.09), *p* < 0.03), and uric acid (OR = 0.55 (95% CI: 0.33–0.92), *p* < 0.03) remained significant predictors in the model of “cumulative global platelet reactivity,” while leukocyte count and free SH groups remained a little beyond significance (OR = 4.88 (95% CI: 0.82-29.01), *p* = 0.079; OR = 1.25 (95% CI: 0.98-1.59), *p* = 0.067, respectively) (*P*_Hosmer-Lemeshow_ = 0.744). Thus, blood platelet morphological parameters appeared as the best predictors of higher platelet reactivity, while the concentration of uric acid predicted the best lower platelet reactivity, also upon the adjustment of the models for sex and age, and other explanatory variables that demonstrated significant individual impacts.

**Figure 1:**
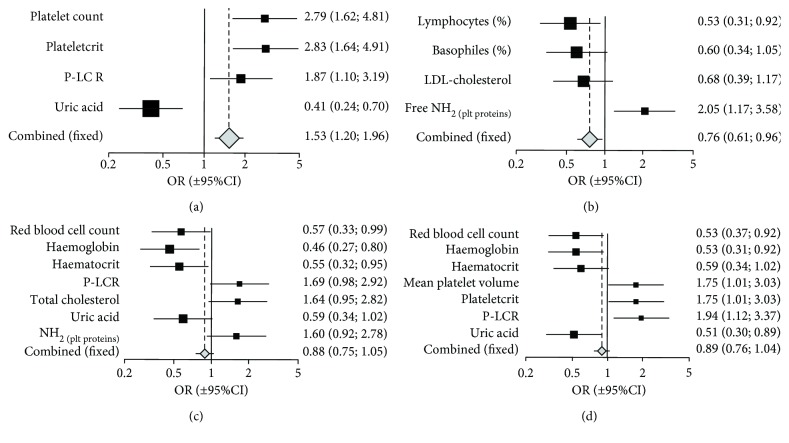
Cochrane odds ratio plot for selected blood morphology and biochemical parameters as risk factors for elevated aggregation (a), activation (b), or reactivity (c, d) of blood platelets in elderly persons. Fixed effects Mantel-Haenszel pooled odds ratio was OR = 1.531 (95% CI = 1.198-1.955, *p* < 0.001) for “cumulative platelet reactivity_aggregation' (a), OR = 0.817 (95% CI = 0.633-1.054, *p* = 0.133) for “cumulative platelet activation” (flow cytometry) (b), OR = 0.947 (95% CI = 0.792-1.132, *p* = 0.579) for “cumulative platelet reactivity” (flow cytometry) (c), and OR = 0.934 (95% CI = 0.789-1.105, *p* < 0.448) for “cumulative global platelet reactivity” (aggregometry and flow cytometry) (d). The sets of data include the explanatory variables showing significant associations with platelet activation (flow cytometry) or a global platelet reactivity (aggregation and flow cytometry). The grouping variable was assigned based on the van der Waerden normalized values of platelet activation (cumulated through surface membrane antigen expressions) or the van der Waerden normalized values of platelet response to agonists (cumulated through the used agonists, estimated surface membrane antigen expressions and the assay employed to monitor platelet reactivity), which were dichotomized to produce the opposed ranks equal to either 0 (when being below or equal to median range; ≤Me, rank 0) or 1 (when being above median range; >Me, rank 1). OR values are presented on a logarithmic scale; 95% confidence intervals of odds ratios are displayed as solid squares with horizontal lines (for a given parameter; the sizes of squares are relevant to sample sizes of strata) or as a diamond (for pooled explanatory variables) with a central vertical dotted line denoting the pooled odds ratio itself.

**Figure 2:**
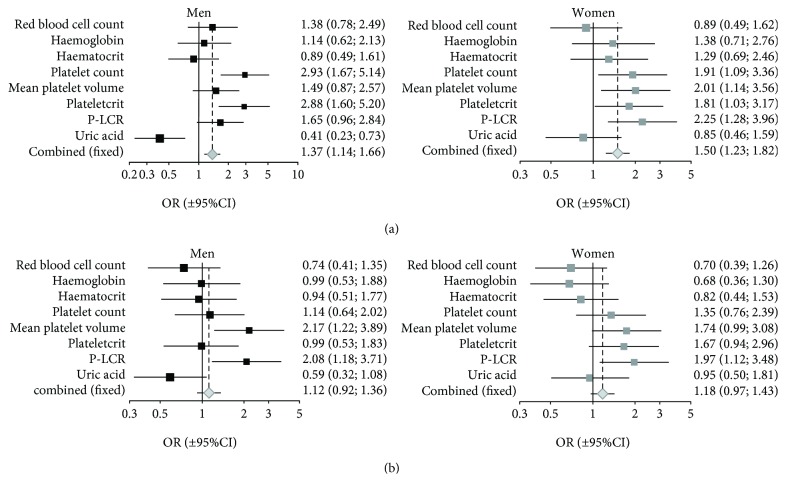
Cochrane odds ratio plot for selected blood morphology and biochemical variables as risk factors (stratified for sex) for elevated platelet aggregation (a) or cumulative global reactivity (b) of blood platelets in elderly persons. Fixed effects Mantel-Haenszel pooled odds ratio was OR = 1.180334 (95% CI = 1.000-1.393, *p* = 0.005) for “cumulative platelet reactivity_aggregation” in men and OR = 1.355 (95% CI = 1.140-1.611, *p* < 0.001) in women (a) and OR = 1.119 (95% CI = 0.920-1.362, *p* = 0.279) for “cumulative global platelet reactivity” in men and OR = 1.175 (95% CI = 0.966-1.429, *p* = 0.114) in women (b). The sets of data include the explanatory variables showing significant associations with platelet aggregation or a global platelet reactivity (aggregation and flow cytometry). The grouping variable was assigned based on the van der Waerden normalized values of platelet reactivity (cumulated through agonists used for aggregation, “cumulative platelet reactivity_aggregation”) or the van der Waerden normalized values of platelet response to agonists (cumulated through the used agonists, estimated surface membrane antigen expressions, and the assay employed to monitor platelet reactivity, “cumulative global platelet reactivity”), which were further dichotomized to produce the opposed ranks equal to either 0 (when being below or equal to median range; ≤Me, rank 0) or 1 (when being above median range; >Me, rank 1). OR values are presented on a logarithmic scale; 95% confidence intervals of odds ratios are displayed as solid squares with horizontal lines (for a given parameter; the sizes of squares are relevant to sample sizes of strata) or as a diamond (for pooled explanatory variables) with a central vertical dotted line denoting the pooled odds ratio itself.

The graphical illustration of the above-described analysis may be Figures 1 and 2 that show the Cochrane odds ratio plots for selected blood morphology (set 1), metabolic (set 2), and free radical markers (set 3) parameters as potential risk factors underlying the elevated activation of circulating blood platelets or altered platelet response to agonists in elderly persons. The dependent variable was either “cumulative platelet reactivity_aggregation” (cumulated through agonists), “cumulative platelet activation” (superimposed through the surface membrane receptor), or “cumulative global platelet reactivity” (cumulated through agonists, surface membrane antigen, and the assay of platelet function). Thus, estimated cumulative measures of platelet activation/reactivity were dichotomized according to a median value in such a way that the values below or equal to median were classified as “lower platelet activation/reactivity” and ranked 0, while the values above median were ranked 1 and referred to as “higher platelet activation/reactivity.” *Per analogiam*, the continuous explanatory variables, describing the selected blood morphology and metabolic or free radical markers parameters and showing significant associations with blood platelet activation/reactivity, were dichotomized in the same way. The 95% confidence interval horizontal line distant from OR = 1.0 (vertical solid line) denotes the significant effect of a given variable on platelet activation/reactivity.

For the “cumulative platelet reactivity_aggregation,” PLT, PCT, P-LCR, and uric acid (OR_combined_ = 1.53; 95% CI: 1.20-1.96; *p* < 0.02) appeared as the most significant contributors to the pooled odds ratio (Figure 1(a)). For the “cumulative platelet activation” (flow cytometry), Lymph%, Baso%, and NH_2_-free plt (OR_combined_ = 0.76; 95% CI: 0.61-0.96; *p* < 0.05) contributed to the greatest extent to the significant value of the pooled odds ratio (Figure 1(b)). For the “cumulative platelet reactivity” monitored with flow cytometry, RBC, HGB, and HCT contributed mostly to the pooled OR (OR_combined_ = 0.88; 95% CI: 0.75-1.05; *p* = 0.089); P-LCR, Neu%, uric acid, and total cholesterol were at the borderline of significance (Figure 1(c)). For the “cumulative global platelet reactivity” in response to agonists RBC, HGB, MPV, PCT, P-LCR, and uric acid contributed most significantly, although oppositely, to the pooled OR (OR_combined_ = 0.89; 95% CI: 0.76-1.04; *p* = 0.087) (Figure 1(d)). Similar analyses were performed after stratification for sex (Figures 2(a) and 2(b)). Likewise, the dependent variable was either “cumulative platelet reactivity_aggregation” (cumulated through agonists) or “cumulative global platelet reactivity” (cumulated through agonists, surface membrane antigen and the assay of platelet function). The estimated cumulative measures of platelet reactivity were dichotomized according to a median value in such a way that the values below or equal to median were classified as “lower platelet reactivity” and ranked 0, while the values above median were ranked 1 and referred to as “higher platelet reactivity”. *Per analogiam*, the continuous explanatory variables, describing the selected blood morphology and metabolic or free radical markers parameters and showing significant associations with blood platelet reactivity, were dichotomized in the same way. As in the case of Figure 1, the 95% confidence interval horizontal line distant from OR = 1.0 (vertical solid line) denotes the significant effect of a given variable on platelet activation/reactivity. For the “cumulative platelet reactivity_aggregation,” PLT and PCT appeared much more significant contributors in men than in women. Otherwise, both MPV and P-LCR contributed significantly in women but not in men. Uric acid and free amino groups in plasma proteins contributed very significantly in men, but remain beyond significance in women (Figure 2(a)). For the “cumulative global platelet reactivity” in response to agonists merely MPV in men, P-LCR in both sexes and uric acid in men appeared significant in contributing to higher or lower global platelet reactivity (Figure 2(b)).

Further, in order to answer the question of which analyzed confounding/coexplanatory variables contribute in the highest extent to the discrimination between lower and higher blood platelet reactivity, we used a linear discriminant analysis. Dichotomized values of “cumulative platelet reactivity_aggregation,” “cumulative platelet activation_flow cytometry,” and “cumulative global platelet reactivity” were used as grouping variables. Our rationale was based on the values of the partial Wilk's lambda: the lower the value, the lower likelihood of the removing of a given variable from a model, and hence, the higher contribution of such a variable to the discrimination between lower and higher reactivity (based on the significance calculated for Mahalanobis' distances). In general, based on the values of the partial Wilk's lambda, for all patients together, we revealed that platelet count (*λ*_partial Wilks_ = 0.982, *p* < 0.04), P-LCR (*λ*_partial Wilks_ = 0.977, *p* < 0.02), and MPV (*λ*_partial Wilks_ = 0.981, *p* < 0.04) were the most discriminative variables amongst blood morphology parameters for the dichotomized *A*_max_ cumulated through agonists, whereas uric acid (*λ*_partial Wilks_ = 0.954, *p* < 0.001) and triglycerides (*λ*_partial Wilks_ = 0.981, *p* < 0.04) stood out amongst metabolic variables, and free thiol groups in platelet proteins (*λ*_partial Wilks_ = 0.981, *p* < 0.04) and superoxide radical generation in platelets in the presence of Hcy (*λ*_partial Wilks_ = 0.961, *p* < 0.02) amongst the variables describing free radical generation (the forward stepwise approach). For the dichotomized global reactivity cumulated through agonists, surface membrane antigens, and assays, platelet count (*λ*_partial Wilks_ = 0.937, *p* < 0.0001), MPV (*λ*_partial Wilks_ = 0.931, *p* < 0.0001), and haemoglobin (*λ*_partial Wilks_ = 0.972, *p* < 0.02) were most discriminative amongst the variables of blood morphology, uric acid (*λ*_partial Wilks_ = 0.921, *p* < 0.0001) and triglycerides (*λ*_partial Wilks_ = 0.973, *p* < 0.02) amongst metabolic parameters, whereas free thiol groups in platelet proteins (*λ*_partial Wilks_ = 0.946, *p* < 0.0005) and superoxide radical generation in platelets in the presence of Hcy (*λ*_partial Wilks_ = 0.974, *p* < 0.015) amongst the variables describing free radical generation. Lastly, for the dichotomized “cumulative platelet activation flow cytometry” cumulated through antigens P-LCR (*λ*_partial Wilks_ = 0.982, *p* < 0.05) and MPV (*λ*_partial Wilks_ = 0.982, *p* < 0.05), LDL cholesterol (*λ*_partial Wilks_ = 0.965, *p* < 0.01) and vWF (*λ*_partial Wilks_ = 0.982, *p* < 0.05), as well as superoxide radical generation in platelets in the presence of Hcy (*λ*_partial Wilks_ = 0.981, *p* < 0.04) and TOS (*λ*_partial Wilks_ = 0.973, *p* < 0.02) discriminated to the highest extent the individuals with lower and higher activation of circulating blood platelets.

Overall, platelet morphology parameters, uric acid, free thiol groups in platelet proteins, and superoxide radical generation in platelets appeared as the variables of the highest discriminative potential for patients with either “lower” or “higher” platelet reactivity.

Canonical analysis was another approach undertaken to answer the question of which of the studied variables contributed to the highest extent to the significant association between blood platelet reactivity and the sets of (i) blood morphology parameters, (ii) metabolic parameters, and (iii) free radical generation parameters. The idea was to build up a canonical (common) variable for each of the sets of variables and to estimate the associations between various canonical variables (describing various sets of variables) in order to figure out which of the examined variables contribute to the formation of the strongest associations. The set of platelet reactivity variables consisted of the *A*_max_ values recorded in the presence of AA, collagen, and ADP (“cumulative reactivity_aggregation”) or the expression values of P-selectin and the active GPIIb/IIIa recorded in response to AA or collagen (“cumulative platelet reactivity_flow cytometry”). The remaining sets consisted the variables arranged as described above. We were interested to figure out which of the possible sets of independent (explanatory) variables explain the maximal variability in the set of dependent variables (aggregometry and flow cytometry parameters of reactivity).  Table 3 presents the canonical correlation coefficients between the canonical variables describing platelet reactivity and the canonical variables describing blood morphology parameters, metabolic parameters, or free radical generation parameters. Based on the estimates of the Wilks' lambda, HGB and HCT, as well the variables describing blood platelet morphometry, appeared the highest contributors to platelet reactivity monitored with whole blood impedance aggregometry, while metabolic parameters (Hcy and cholesterol in lipoprotein fractions) contributed to the highest extent to platelet reactivity monitored with flow cytometry. Regardless of whether aggregometry or flow cytometry was employed for the monitoring of blood platelet reactivity, the variables describing blood platelet morphometry, cholesterol, lipid peroxides in platelets, and superoxide anion generation in the presence of Hcy appeared the most significant contributors to shaping of platelet reactivity.

## 4. Discussion

In our paper, we focus mainly on blood platelet-derived cardiovascular risk, and hence, when referring to the term “platelet cardiovascular risk,” we understand specifically platelet reactivity, i.e., the readiness of blood platelets to response to agonists upon a stimulation of haemostatic system, in a perspective leading to atherosclerosis and ischaemic events. It would be desirable to get much more profound and multifaceted information about factors responsible for platelet quiescence and activation in order to monitor either the progression or regression of cardiovascular risk with the use of supposedly newly elaborated algorithms, involving easily measured diagnostic parameters, like those describing blood morphology or serum biochemistry. We have searched which of these parameters can be significant explanatory variables defining blood platelet functioning in the investigated group of older people. We have intentionally segregated the explanatory variables of interest into three sets of variables: (a) those concerning blood morphology (counts, volumes, cellcrits, and morphological indices), (b) those referring serum biochemistry (including lipids, carbohydrates, fatty acid or nucleic acid metabolites, and endothelial markers), and (c) redox balance indicators. Using both simple and multivariate statistical analyses, we made an attempt to define which of the sets of explanatory variables contribute to the greatest extent to the “shaping” of blood platelet activation and reactivity in the investigated group of older individuals. According to our analysis, the most potent modulators of platelet reactivity are morphological parameters, amongst which the variables describing blood platelets and red blood cells appeared as the leading ones. Further, amongst biochemical factors in blood plasma, uric acid should be seen as a compound showing a strong impact on platelet reactivity. Otherwise, it seems that oxidative stress variables contribute to platelet reactivity to the lowest extent. Amongst oxidative parameters investigated in our study, only free thiols of blood platelet proteins, platelet lipid peroxides, and the generation of superoxide anion by platelets appeared the significant contributors to platelet functional state.

Blood platelets can be considered as vehicles of the solid phase of a coagulation cascade. In this point of view, platelet membranes serve as a molecular platform for the anchoring of haemostatic factors. Moreover, platelets act as “transporters” of coagulation inducers and propagators, physiologically stored in intraplatelet vesicles and released in a controlled manner upon platelet activation. In a course of subsequent episodes of activation, platelets degranulate and loose the contents of their dense and alpha granules. It seems understandable then that frequently releasing platelets gradually become smaller and less functional. The fate of such often stimulated blood platelets may be diagnostically tracked by the monitoring of mean platelet volume (MPV), an important hallmark of a long-term intensity of platelet response, in such a sense that bigger platelets (characterized by higher MPV values in blood morphology analysis) bring about an increased membrane surface for coagulation and also higher concentrations of haemostatic factors secreted from bigger vesicles formed in a cellular compartment surrounded by platelet membrane. In this study, we confirm that also in older adults, morphological parameters of blood platelets, and especially, mean platelet volume (MPV), may be regarded as significant markers of atherogenesis, thrombogenicity, and a risk factor for adverse cardiovascular events [2, 41, 42].

Larger MPV values are associated with some hallmarks of prothrombotic states, like a weaker platelet response to antiplatelet drugs [43, 44]. Since relationship has been claimed between MPV and platelet reactivity, it might be assumed that bigger platelets are potentially more reactive cells [45]. Our outcomes presenting positive association between MPV and platelet aggregability are supportive for the abovementioned observations.

According to our results, a very significant contribution of the morphometric characteristics of blood platelets, with special regard to MPV, but also PLT and P-LCR, imply that it may be largely blood platelet morphological features that shape out platelet responsiveness to agonists, which in turn, thus underlying the risk of atherosclerosis development.

Our present study demonstrates that apart from platelet parameters of blood morphology, the multifactorial “bunch” of factors affecting platelets activation and reactivity also includes some other molecules protective for platelets, for instance, uric acid. In our analysis, this final product of purine metabolism appeared a very significant predictor of lower cumulative platelet reactivity, despite the lack of a significant difference in the concentrations of uric acid between the subgroups of subjects with lower and higher cumulative platelet reactivity. Importantly, a high significance of uric acid was maintained also upon adjustment for several confounding factors, including sex, serum glucose, or lipids. In recent years, uric acid raises the interest of researchers as a molecule potentially involved in atherogenesis. The evidence presented herein confirms that uric acid is an important player in the cardiovascular game. It should be emphasized that our present study is one of the first to indicate the possibility that uric acid is able to affect atherogenesis by affecting platelets. This observation, however, is in a clear contradiction to the very first reports [21, 46] and also more recent one [47], showing the lack of relationships between uricaemia and blood platelet function. Maybe, the reason for the occurring discrepancy concerns differences in the characteristics of studied populations. Since we have shown that serum uricaemia significantly affects platelet activation and reactivity, it would seem tempting to further estimate whether uric acid may impact platelet activation and reactivity only under basal conditions or it can also modulate platelet sensitivity to antiplatelet drugs. On the basis of available literature reports, it seems that platelet sensitivity to acetylsalicylic acid and clopidogrel or ticagrelor is not directly affected by uricaemia [48, 49]. Interestingly, both acetylsalicylic acid [50] and ticagrelor [49] increase serum levels of uric acid, which—together with the associations between uricaemia and platelet activation/reactivity evaluated herein—favors a possibly new pathway of antiplatelet action of these drugs. On the other hand, it has to be recalled that the outcomes recently reported by Barbier et al. [48] and Nardin et al. [49] make such a bold suggestion questionable. Nevertheless, we can suggest that serum uricaemia stays in a significant negative association with platelet responsiveness to agonists.

This overall picture of the factors shaping atherogenic risk may be even more complicated, when including red blood cells as another significant contributor to enhanced platelet reactivity. Erythrocytes are involved in modulating of platelet aggregation in different ways, of which the impacts of nitric oxide (NO) and ADP are worth of mentioning. NO, produced in RBC membrane and cytoplasm by endothelial-type nitric oxide synthase (eNOS) [51, 52], contributes to the impeding of platelet aggregability and has been shown to exert a significant antiaggregatory effect on blood platelets [52]. Interestingly, older erythrocytes are more efficient producers of NO in comparison to their younger counterparts [53]. Otherwise, huge amounts of ADP stored in red blood cells might certainly play a role in facilitating platelet aggregation in the course of thrombus formation [54]. We are not aware to which extents such a compensatory (in the case of NO) or propelling mechanism (in the case of ADP) may really occur and function in older adults; however, our findings on the negative associations between the number of RBC and haematocrit with platelet aggregability demonstrated in this study, rather support the predominating effects of RBC-derived NO than ADP. Thus, as far as we regard erythrocytes as a potent source of antiplatelet NO in a peripheral blood, we demonstrate herein that lower number of erythrocytes may lead to higher blood platelet activation and reactivity.

Hcy, which is known as a strong enhancer of platelet reactivity *in vitro* [33], herein appeared as rather a nonsignificant contributor to platelet activation and reactivity. Evidently, Hcy seems to be not involved in platelet reactivity *in vivo* to a considerable extent, and this may explain—partly at least—why some interventions aimed at Hcy lowering do not bring any spectacular and significant improvements in cardiovascular risk [55]. Our results appear to confirm rather a weak contribution of Hcy to augmenting of platelet activation and reactivity, as risk factors relevant to cardiovascular risk. Moreover, results showing that Hcy-induced, but not basal, generation of superoxide anion by blood platelets *in vitro* further documents that when Hcy-dependent changes of platelet-dependent haemostasis are considered, there is a great risk of generating conflicting results originating from *in vitro* and ex vivo measurements. The markers of endothelial function chosen by us for this study remained, in general, with no significant association with the tested parameters of platelet reactivity, which implies that they are not useful predictors of platelet functional state. Similarly, a plethora of different redox markers of platelets and plasma remained with no significant relation to platelet activation and reactivity, despite the fact that some of them have been favorably used as markers of platelet response to different stimuli in simplified *in vitro* models [56, 57].

As it can be seen in Table 1, women exhibited higher platelet reactivity than men. This commonly noted sex dependence of platelet reactivity is distinct both for younger [58] and for older populations [59]. Since we were aware of the importance of sex in the shaping of platelet reactivity, we reconducted our search of the factors mostly contributing to the functional state of blood platelets also upon stratification for sex. Also, in such an approach, morphological parameters of blood platelets took the first place in the order of factors shaping out platelet reactivity. Thus, it seems plausible that sex-dependent intrinsic factors govern platelet reactivity, probably at the level of thrombopoiesis, when morphology of blood platelets is programmed [60, 61].

## 5. Conclusions

In summary, according to the outcomes of different statistical approaches employed in this study, we declare that the most significant in their contribution to platelet reactivity are platelet morphology, plasma uricaemia, and erythrocyte morphology. In the light of these findings, it becomes clearer now that the prediction of cardiovascular risk based on simple measurements of single, isolated variables, like platelet morphology or selected biochemical predictors, may be misleading and may result in the misestimation, and more even so, when referring to simplified *in vitro* models of mutual platelet-biochemical interactions amongst various blood constituents.

## Figures and Tables

**Table 1:** Blood morphology, serum biochemistry, and markers of platelet reactivity of investigated subjects.

Variable	Both sexes *n* = 251	Females *n* = 125	Males *n* = 126
White blood cells [WBC] (10^3^/mm^3^)	5.8 (5.0; 6.9)	5.6 (4.9; 6.6)	6 (5.2; 7.1)^U^^∗^
Red blood cells [RBC] (10^6^/mm^3^)	4.5 ± 0.4	4.3 ± 0.3	4.6 ± 0.4^U††^
Haemoglobin [HGB] (g/dl)	13.8 (13.0; 14.6)	13.3 (12.7; 13.8)	14.4 (13.8; 15.1)^U††^
Haematocrit [HCT] (%)	39.9 (37.6; 41.7)	38.6 (36.9; 40.0)	41.1 (39.5; 43)^U††^
Mean corpuscular volume (fl)	88.3 (86.2; 91.3)	88.4 (86.4; 90.3)	88.1 (86.2; 91.7)
Mean corpuscular haemoglobin (pg)	30.7 (29.8; 31.6)	30.5 (29.6; 31.3)	30.9 (30; 31.9)^U†^
Mean corpuscular haemoglobin concentration (g/dl)	34.7 (34.1; 35.3)	34.3 (33.9; 34.9)	35 (34.5; 35.6)^U††^
Blood platelets [PLT] (10^3^/mm^3^)	212.5 (181.0; 243.0)	225.5 (198.5; 265.0)	196 (168; 226)^U††^
Mean platelet volume [MPV] (*μ*m^3^)	11.3 (10.8; 12.1)	11.3 (10.8; 12.1)	11.4 (10.7; 12.1)
Plateletcrit [PCT] (%)	0.24 (0.21;0.28)	0.26 (0.23; 0.29)	0.22 (0.19; 0.25)^U††^
Platelets distribution width [PDW] (fl)	13.6 (12.5; 15.6)	13.5 (12.6; 15.6)	13.9 (12.3; 15.7)
Platelets large cell ratio [P-LCR] (%)	36.2 ± 7.7	36.6 ± 8.0	36.0 ± 7.5
Lymphocytes (10^3^/mm^3^)	1.96 (1.6; 2.4)	1.96 (1.6; 2.3)	1.94 (1.6; 2.4)
Monocytes (10^3^/mm^3^)	0.53 (0.45; 0.66)	0.50 (0.40; 0.58)	0.57 (0.48; 0.72)^U††^
Neutrophils (10^3^/mm^3^)	3.09 (2.56; 3.87)	3.00 (2.50; 3.76)	3.18 (2.66; 3.89)
Eosinophils (10^3^/mm^3^)	0.15 (0.10; 0.22)	0.13 (0.09; 0.18)	0.16 (0.11; 0.25)^U#^
Basophiles (10^3^/mm^3^)	0.03 (0.02; 0.03)	0.03 (0.02; 0.03)	0.03 (0.02; 0.03)
Total cholesterol (mg/dl)	207.1 (137.1; 237.0)	222 (183.5; 253.1)	187.2 (166.4; 219)^U††^
Triglycerides (mg/dl)	111.5 (77.8; 163.0)	111.7 (76.8; 159.8)	111.2 (78.4; 167.1)
HDL cholesterol (mg/dl)	48.4 (40.9; 59.1)	54.1 (45.7; 64.3)	44.3 (38.7; 51.1)^U††^
LDL cholesterol (mg/dl)	131.2 (103.3; 156.3)	(108.8; 170.8)	115.6 (100.1; 146.5)^U††^
Glucose (mg/dl)	99.2 (91.4; 108.5)	96.3 (89.3; 105.8)	101 (93.8; 113.1)^U†^
Uric acid (mg/dl)	4.9 ± 1.2	4.3 ± 1.2	5.4 ± 1.1^T††^
Homocysteine [Hcy] (*μ*mol/l)	14.7 (12.5; 17.2)	14.2 (12.3; 16.2)	15.7 (13.1; 18.1)^U†^
*A*_max_ __AA_	127.9 (109.1; 145.1)	134.6 (117.4; 152.4)	123.2 (101.3; 137.3)^U††^
*A*_max_ __COL_	152.6 (132.4; 176.6)	158.3 (137.3; 184.9)	146.8 (121.9; 166.5)^U^^∗^
*A*_max_ __ADP_	122.3 (103.9; 140.3)	128.3 (114.8; 144.8)	113 (94; 131)^U††^

Variables are presented as means ± SD and medians with interquartile ranges or percent fractions of whole groups of investigated patients. Comparisons between men and women performed with the use of the unpaired Student *t*-test (^T^), or the Mann-Whitney *U* test (^U^). ^∗^*p* ≤ 0.05; ^#^*p* < 0.01; ^†^*p* < 0.001; ^††^*p* < 0.001. AA: arachidonic acid; ADP: adenosine diphosphate; *A*_max_: maximal platelet aggregation; COL: collagen; HDL: high-density lipoprotein; LDL: low-density lipoprotein.

**Table 2:** Blood morphology and metabolic and free radical generation parameters in subjects with lower and higher blood platelet reactivity.

Variable	Subjects with lower platelet reactivity *n* = 126	Subjects with higher platelet reactivity *n* = 125	*p* value
White blood cells (10^3^/mm^3^)	5.7 (4.9; 6.9)	5.8 (5.2; 6.9)	<0.05
Red blood cells (10^6^/mm^3^)	4.5 ± 0.4	4.5 ± 0.4	n.s.
Haemoglobin (g/dl)	14.0 (13.1; 14.7)	13.7 (12.9; 14.3)	n.s.
Haematocrit (%)	40.1 (38.2; 42.0)	39.6 (37.6; 41.2)	n.s.
Blood platelets (10^3^/mm^3^)	202.4 (169.4; 231.4)	223.1 (191.1; 267.2)	<0.01
Mean platelet volume (*μ*m^3^)	11.2 (10.6; 12.0)	11.4 (10.9; 12.4)	<0.01
Plateletcrit (%)	0.2 (0.3; 0.3)	0.3 (0.2; 0.3)	<<0.0001
Platelet distribution width (fl)	13.3 (12.2; 15.2)	13.9 (12.7; 16.3)	<0.01
Platelet-large cell ratio (%)	34.6 ± 7.4	37.7 ± 7.9	<0.01
Lymphocytes (10^3^/mm^3^)	1.9 (1.6; 2.3)	2.0 (1.6; 2.4)	n.s.
Monocytes (10^3^/mm^3^)	0.5 (0.4; 0.7)	0.5 (0.4; 0.7)	n.s.
Neutrophils (10^3^/mm^3^)	3.0 (2.5; 3.8)	3.1 (2.7; 3.9)	<0.05
Total cholesterol (mg/dl)	203.6 (172.6; 227.5)	207.9 (177.3; 240.8)	n.s.
Triglycerides (mg/dl)	108.3 (76.7; 154.1)	111.8 (78.4; 167.0)	n.s.
HDL cholesterol (mg/dl)	47.7 (40.9; 57.6)	48.4 (42.5; 58.4)	n.s.
LDL cholesterol (mg/dl)	124.7 (101.1; 151.5)	131.5 (103.3; 161.1)	n.s.
Glucose (mg/dl)	100.2 (92.1; 110.8)	97.1 (90.8; 107.6)	n.s.
Uric acid (mg/dl)	5.1 (4.2; 5.8)	4.4 (3.8; 5.4)	n.s.
Homocysteine (*μ*mol/l)	14.9 (12.5; 17.8)	14.5 (12.5; 16.9)	n.s.
von Willebrand factor (*μ*g/ml)	5.5 (4.9; 6.1)	5.6 (4.9; 6.2)	n.s.
Vascular cell adhesion protein 1 (ng/ml)	269.4 (248.1; 293.9)	273.7 (249.2; 297.1)	n.s.
Intracellular adhesion molecule (ng/ml)	210.1 (203.4; 217.7)	210.0 (202.4; 216.2)	n.s.
*A*_max_ __AA_	108.9 (84.6; 123.9)	145.1 (132.6; 158.6)	<<0.0001
*A*_max_ __COL_	134.6 (110.6; 147.0)	173.9 (157.4; 190.4)	<<0.0001
*A*_max_ __ADP_	105.3 (90.7; 115.8)	138.5 (125.7; 152.9)	<<0.0001
“Cumulative platelet reactivity_aggregation”	-1.5 (-2.4; -0.7)	1.9 (1.0; 2.8)	<<0.0001
P-selectin _resting platelets_	5.9 (2.2; 15.4)	6.3 (2.3; 13.4)	n.s.
Activated GPIIb/IIIa_resting platelets_	7.0 (2.0; 18.7)	7.1 (1.9, 27.2)	n.s.
“Cumulative platelet activation”	0.004 (-1.6; 1.2)	-0.09 (-1.2; 1.0)	n.s.
P-selectin_AA_	29.5 (14.0; 45.6)	37.4 (17.1; 57.8)	<0.05
P-selectin_COL_	26.8 (13.4; 44.1)	30.3 (12.1; 53.1)	n.s.
“Cumulative P-selectin expression'	-0.24 (-1.1; 0.8)	0.4 (-1.4; 1.6)	n.s.
Activated GPIIb/IIIa_AA_	33.6 (16.8; 51.9)	36.5 (21.0; 54.7)	n.s.
Activated GPIIb/IIIa_COL_	34.6 (17.2; 55.0)	37.1 (17.7; 56.9)	n.s.
“Cumulative activated GPIIb/IIIa expression”	-0.05 (-1.4; 1.07)	0.07 (-1.0; 1.1)	n.s.
“Cumulative global platelet reactivity”	-2.1 ± 3.2	2.2 ± 3.2	<<0.0001
Free amino groups_platelet proteins_ (nmol/*μ*g of protein)	0.2 (0.05; 1.7)	0.2 (0.05; 2.7)	n.s.
Free amino groups_plasma proteins_ (mmol/mg of protein)	17.6 (11.0; 26.3)	16.1 (10.8; 25.7)	n.s.
Thiol groups_platelet proteins_ (*μ*mol/*μ*g of protein)	2.9 (2.1; 22.1)	2.9 (1.8; 88.3)	n.s.
Thiol groups_plasma proteins_ (*μ*mol/*μ*g of protein)	0.03 (0.02; 0.05)	0.03 (0.02; 0.04)	n.s.
Lipid peroxides_plt_ (nmol/*μ*g of protein)	1.04 (0.37-23.76)	1.4 (0.6; 29.6)	n.s.
Lipid peroxides_plasma_ (mmol/l)	0.2 (0.005; 1.2)	0.3 (0.05; 1.4)	n.s.
O_2_^−^_plt_	0.5 (0.2; 4.6)	0.4 (0.1; 5.6)	n.s.
O_2_^−^_plt Hcy_	0.6 (0.2; 6.3)	0.5 (0.1; 7.3)	n.s.

Variables are presented as means ± SD or medians with interquartile ranges. The cumulative normal scores through the used agonists (AA, COL, and ADP) for whole blood aggregometry (*A*_max_) were dichotomized according to the median in such a way that the values below or equal to median (≤Me, rank 0) corresponded to the “lower blood platelet reactivity”, while the values above median (>Me, rank 1) corresponded to the “higher blood platelet reactivity,” and such categorical measures of platelet reactivity were accepted as the grouping variable for the comparison of subjects with lower platelet reactivity and subjects with higher platelet reactivity. Nontransformed data (means and SD or medians and interquartile ranges) are shown for lower and higher platelet reactivity groups of individuals. All the variables (after the Box-Cox transformations when the assumptions of normal distribution and/or homogeneity of variances were violated) were compared upon the adjustment for age and sex with the bootstrap-boosted (10000 iterations) analysis of covariance (ANCOVA). AA: arachidonic acid; ADP: adenosine diphosphate; *A*_max_: maximal aggregation of blood platelets; COL: collagen; free amino groups_plasma proteins_/free amino groups_platelet proteins_: the content of free amino groups in plasma/platelet proteins; Hcy: homocysteine; HDL: high-density lipoprotein; LDL; low-density lipoprotein; n.s.: nonsignificant; O_2_^−^_plt_/O_2_^−^_plt Hcy_: generation of superoxide anion in platelets in the absence/presence of Hcy; plt: blood platelets; “cumulative platelet reactivity_aggregation”: platelet reactivity cumulated through agonists (AA, COL, or ADP) and monitored with whole blood impedance platelet aggregometry; “cumulative platelet activation”: the expression values of P-selectin and the activated GPIIb/IIIa recorded in circulating (resting) platelets cumulated through antigens (P-selectin, activated GPIIb/IIIa) and monitored with flow cytometry; “cumulative P-selectin expression”: P-selectin expression on platelet surface membrane cumulated through agonists (AA and collagen) and monitored with flow cytometry; “cumulative active GPIIb/IIIa expression”: activated GPIIb/IIIa expression on platelet surface membrane cumulated through agonists (AA and collagen) and monitored with flow cytometry; “cumulative global platelet reactivity”: platelet reactivity cumulated through agonists (AA, COL, and ADP) and assays (aggregometry: *A*_max_ in the presence of AA, collagen, or ADP and flow cytometry: the expression values of P-selectin and the activated GPIIb/IIIa).

**Table 3:** Canonical correlations between the set of blood platelet reactivity parameters and the sets of blood morphology, metabolism, and free radical generation parameters.

Platelet reactivity (set 1)	Extracted variance (%)	Total redundancy (%)	Explanatory variables (set 2)	Extracted variance (%)	Total redundancy (%)	Canonical correlation	Canonical determination (*R*^2^)	*P*	Wilks' lambda	Best contributors
*Aggregometry*	100.0	26.5	*Blood morphology*	70.3	12.1	0.716	0.513	0.003	0.235	HGB, HCT, MPV, PCT, and P-LCR
*Aggregometry*	100.0	21.4	*Metabolic*	60.9	7.7	0.575	0.330	0.020	0.262	TC, LDL, and UA
*Aggregometry*	100.0	8.4	*Free radical*	100.0	11.7	0.580	0.336	0.100	0.450	POx_plt_, SH_pl_, and O_2plt_
*Flow cytometry*	100.0	17.3	*Blood morphology*	40.6	5.1	0.495	0.245	0.033	0.406	MPV, P-LCR, HCT, PDW, and HGB
*Flow cytometry*	100.0	13.4	*Metabolic*	46.1	6.9	0.522	0.272	0.004	0.366	Hcy, HDL, LDL, and TxB_2_
*Flow cytometry*	100.0	11.7	*Free radical*	69.0	7.9	0.539	0.290	0.006	0.509	POx_plt_ and O_2_^−^_plt_

Rho^2^ is a squared canonical correlation coefficient (canonical determination), which is relevant to variance between canonical variables. Total redundancy is relevant to the variance between the canonical variable for set 1 and the variables of set 2; it shows the representation of a given canonical variable by the explanatory variables of the set 2. Extracted variance is a variance between a given canonical variable and the variables that build it up; it reflects how well a given canonical variable represents a given set of variables. The Wilks' lambda is the parameter reflecting the contribution of a set 2 to the explaining of the variance of a set 1; the lower is the Wilks' lambda, the higher is the contribution. HCT: haematocrit; HDL: high-density lipoproteins; HGB: haemoglobin; Hcy: homocysteine; LDL: low-density lipoproteins; MPV: mean platelet volume; O_2_^−^_plt_: superoxide anion generated by blood platelets in the presence of Hcy; PCT: plateletcrit; PDW: platelet distribution width; P-LCR: platelet-large cell ratio; POx_plt_; lipid peroxides in blood platelets; SH_plt_: free thiol groups of platelet proteins; TC: total cholesterol; UA: uric acid.

## Data Availability

The data used to support the findings of this study are available from the corresponding author upon request.

## References

[1] Jones C. I. (2016). Platelet function and ageing. *Mammalian Genome*.

[2] Chu S. G., Becker R. C., Berger P. B. (2010). Mean platelet volume as a predictor of cardiovascular risk: a systematic review and meta-analysis. *Journal of Thrombosis and Haemostasis*.

[3] Vinholt P. J., Hvas A. M., Frederiksen H., Bathum L., Jørgensen M. K., Nybo M. (2016). Platelet count is associated with cardiovascular disease, cancer and mortality: a population-based cohort study. *Thrombosis Research*.

[4] Shin D. H., Rhee S. Y., Jeon H. J., Park J. Y., Kang S. W., Oh J. (2017). An increase in mean platelet volume/platelet count ratio is associated with vascular access failure in hemodialysis patients. *PLoS One*.

[5] Larsen E., Palabrica T., Sajer S. (1990). PADGEM-dependent adhesion of platelets to monocytes and neutrophils is mediated by a lineage-specific carbohydrate, LNF III (CD15). *Cell*.

[6] Santos M. T., Valles J., Marcus A. J. (1991). Enhancement of platelet reactivity and modulation of eicosanoid production by intact erythrocytes. A new approach to platelet activation and recruitment. *The Journal of Clinical Investigation*.

[7] de Bruijne-Admiraal L. G., Modderman P. W., Von dem Borne A. E., Sonnenberg A. (1992). P-selectin mediates Ca (2+)-dependent adhesion of activated platelets to many different types of leukocytes: detection by flow cytometry. *Blood*.

[8] Henson P. M. (1977). Activation of rabbit platelets by platelet-activating factor derived from IgE-sensitized basophils. Characteristics of the aggregation and its dissociation from secretion. *The Journal of Clinical Investigation*.

[9] Jawień J., Chłopicki S., Gryglewski R. J. (2002). Interactions between human platelets and eosinophils are mediated by selectin-P. *Polish Journal of Pharmacology*.

[10] Vallés J., Santos M. T., Aznar J. (2002). Platelet-erythrocyte interactions enhance α_IIb_β(3) integrin receptor activation and P-selectin expression during platelet recruitment: down-regulation by aspirin ex vivo. *Blood*.

[11] Pawelski H., Lang D., Reuter S. (2014). Interactions of monocytes and platelets implication for life. *Frontiers in Bioscience*.

[12] Lam F. W., Vijayan K. V., Rumbaut R. E. (2015). Platelets and their interactions with other immune cells. *Comprehensive Physiology*.

[13] Madjid M., Fatemi O. (2013). Components of the complete blood count as risk predictors for coronary heart disease: in-depth review and update. *Texas Heart Institute Journal*.

[14] Kreisberg R. A., Kasim S. (1987). Cholesterol metabolism and aging. *The American Journal of Medicine*.

[15] Strandberg T. E., Tilvis R. S., Pitkala K. H., Miettinen T. A. (2006). Cholesterol and glucose metabolism and recurrent cardiovascular events among the elderly: a prospective study. *Journal of the American College of Cardiology*.

[16] Prociuk M. A., Edel A. L., Richard M. N. (2008). Cholesterol-induced stimulation of platelet aggregation is prevented by a hempseed-enriched diet. *Canadian Journal of Physiology and Pharmacology*.

[17] Wang N., Tall A. R. (2016). Cholesterol in platelet biogenesis and activation. *Blood*.

[18] May J., Loesche W., Heptinstall S. (1990). Glucose increases spontaneous platelet aggregation in whole blood. *Thrombosis Research*.

[19] Brewer R. A., Gibbs V. K., Smith D. L. (2016). Targeting glucose metabolism for healthy aging. *Nutrition and Healthy Aging*.

[20] Sudic D., Razmara M., Forslund M., Ji Q., Hjemdahl P., Li N. (2006). High glucose levels enhance platelet activation: involvement of multiple mechanisms. *British Journal of Haematology*.

[21] Ciompi M. L., De Caterina R., Bertolucci D., Bernini W., Michelassi C., L'Abbate A. (1983). Uric acid levels and platelet function in humans. An in-vivo ex-vivo study. *Clinical and Experimental Rheumatology*.

[22] Feig D. I., Kang D. H., Johnson R. J. (2008). Uric acid and cardiovascular risk. *The New England Journal of Medicine*.

[23] Verdoia M., Barbieri L., Schaffer A. (2014). Impact of diabetes on uric acid and its relationship with the extent of coronary artery disease and platelet aggregation: a single-centre cohort study. *Metabolism Clinical and Experimental*.

[24] Karolczak K., Watala C. (2017). The significance of the interplay between blood platelets and homocysteine in the pathogenesis of coronary artery disease. *Coronary Artery Disease*.

[25] Bonomini F., Tengattini S., Fabiano A., Bianchi R., Rezzani R. (2008). Atherosclerosis and oxidative stress. *Histology and Histopathology*.

[26] Ambrosio G., Golino P., Pascucci I. (1994). Modulation of platelet function by reactive oxygen metabolites. *American Journal of Physiology-Heart and Circulatory Physiology*.

[27] Watt J., Ewart M. A., Greig F. H., Oldroyd K. G., Wadsworth R. M., Kennedy S. (2012). The effect of reactive oxygen species on whole blood aggregation and the endothelial cell-platelet interaction in patients with coronary heart disease. *Thrombosis Research*.

[28] Münzel T., Gori T., Bruno R. M., Taddei S. (2010). Is oxidative stress a therapeutic target in cardiovascular disease?. *European Heart Journal*.

[29] Sołtysik B. K., Kroc Ł., Pigłowska M., Guligowska A., Śmigielski J., Kostka T. (2017). An evaluation of the work and life conditions and the quality of life in 60 to 65 year-old white-collar employees, manual workers, and unemployed controls. *Journal of Occupational and Environmental Medicine*.

[30] Zar J. (1999). *Biostatistical Analysis*.

[31] Karolczak K., Kamysz W., Karafova A., Drzewoski J., Watala C. (2013). Homocysteine is a novel risk factor for suboptimal response of blood platelets to acetylsalicylic acid in coronary artery disease: a randomized multicenter study. *Pharmacological Research*.

[32] Karolczak K., Pieniazek A., Watala C. (2017). Inhibition of glutamate receptors reduces the homocysteine-induced whole blood platelet aggregation but does not affect superoxide anion generation or platelet membrane fluidization. *Platelets*.

[33] Boncler M., Gresner P., Nocun M. (2007). Elevated cholesterol reduces acetylsalicylic acid-mediated platelet acetylation. *Biochimica et Biophysica Acta (BBA) - General Subjects*.

[34] Walkowiak B., Michalak E., Koziolkiewicz W., Cierniewski C. S. (1989). Rapid photometric method for estimation of platelet count in blood plasma or platelet suspension. *Thrombosis Research*.

[35] Watala C., Karolczak K., Kassassir H. (2016). How do the full-generation poly(amido)amine (PAMAM) dendrimers activate blood platelets? Activation of circulating platelets and formation of “fibrinogen aggregates” in the presence of polycations. *International Journal of Pharmaceutics*.

[36] Ando Y., Steiner M. (1973). Sulfhydryl and disulfide groups of platelet membranes. I. Determination of sulfhydryl groups. *Biochimica et Biophysica Acta*.

[37] Sashidhar R. B., Capoor A. K., Ramana D. (1994). Quantitation of epsilon-amino group using amino acids as reference standards by trinitrobenzene sulfonic acid. A simple spectrophotometric method for the estimation of hapten to carrier protein ratio. *Journal of Immunological Methods*.

[38] Bartosz G. (2006). Use of spectroscopic probes for detection of reactive oxygen species. *Clinica Chimica Acta*.

[39] Gresele P., Pignatelli P., Guglielmini G. (2008). Resveratrol, at concentrations attainable with moderate wine consumption, stimulates human platelet nitric oxide production. *The Journal of Nutrition*.

[40] Hosmer D. W., Lemeshow S., Sturdivant R. X. (2013). *Applied Logistic Regression*.

[41] Endler G., Klimesch A., Sunder-Plassmann H. (2002). Mean platelet volume is an independent risk factor for myocardial infarction but not for coronary artery disease. *British Journal of Haematology*.

[42] Makowski M., Smorag I., Makowska J. (2017). Platelet reactivity and mean platelet volume as risk markers of thrombogenesis in atrial fibrillation. *International Journal of Cardiology*.

[43] Kim Y. G., Suh J. W., Yoon C. H. (2014). Platelet volume indices are associated with high residual platelet reactivity after antiplatelet therapy in patients undergoing percutaneous coronary intervention. *Journal of Atherosclerosis and Thrombosis*.

[44] Uzel H., Ozpelit E., Badak O. (2014). Diagnostic accuracy of mean platelet volume in prediction of clopidogrel resistance in patients with acute coronary syndrome. *Anadolu Kardiyoloji Dergisi/The Anatolian Journal of Cardiology*.

[45] Cesari F., Marcucci R., Caporale R. (2008). Relationship between high platelet turnover and platelet function in high-risk patients with coronary artery disease on dual antiplatelet therapy. *Thrombosis and Haemostasis*.

[46] Macfarlane D. G., Slade R., Hopes P. A., Hartog M. H. (1983). A study of platelet aggregation and adhesion in gout. *Clinical and Experimental Rheumatology*.

[47] De Luca G., Secco G. G., Santagostino M. (2012). Uric acid does not affect the prevalence and extent of coronary artery disease. Results from a prospective study. *Nutrition, Metabolism and Cardiovascular Diseases*.

[48] Barbieri L., Verdoia M., Pergolini P. (2016). Uric acid and high-residual platelet reactivity in patients treated with clopidogrel or ticagrelor. *Nutrition, Metabolism and Cardiovascular Diseases*.

[49] Nardin M., Verdoia M., Pergolini P. (2016). Serum uric acid levels during dual antiplatelet therapy with ticagrelor or clopidogrel: results from a single-centre study. *Nutrition, Metabolism and Cardiovascular Diseases*.

[50] Caspi D., Lubart E., Graff E., Habot B., Yaron M., Segal R. (2000). The effect of mini-dose aspirin on renal function and uric acid handling in elderly patients. *Arthritis and Rheumatism*.

[51] Cortese-Krott M. M., Kelm M. (2014). Endothelial nitric oxide synthase in red blood cells: key to a new erythrocrine function?. *Redox Biology*.

[52] Kleinbongard P., Schulz R., Rassaf T. (2006). Red blood cells express a functional endothelial nitric oxide synthase. *Blood*.

[53] Bizjak D. A., Brinkmann C., Bloch W., Grau M. (2015). Increase in red blood cell-nitric oxide synthase dependent nitric oxide production during red blood cell aging in health and disease: a study on age dependent changes of rheologic and enzymatic properties in red blood cells. *PLoS One*.

[54] Alkhamis T. M., Beissinger R. L., Chediak J. R. (1988). Red blood cell effect on platelet adhesion and aggregation in low-stress shear flow. Myth or fact?. *ASAIO Transactions*.

[55] Marcus J., Sarnak M. J., Menon V., Verma S. (2007). Homocysteine lowering and cardiovascular disease risk: lost in translation. *Canadian Journal of Cardiology*.

[56] Olas B., Wachowicz B., Nowak P. (2008). Studies on antioxidant properties of polyphenol-rich extract from berries of Aronia melanocarpa in blood platelets. *Journal of Physiology and Pharmacology*.

[57] Kolodziejczyk J., Olas B., Wachowicz B., Szajwaj B., Stochmal A., Oleszek W. (2011). Clovamide-rich extract from Trifolium pallidum reduces oxidative stress-induced damage to blood platelets and plasma. *Journal of Physiology and Biochemistry*.

[58] Otahbachi M., Simoni J., Simoni G. (2010). Gender differences in platelet aggregation in healthy individuals. *Journal of Thrombosis and Thrombolysis*.

[59] Karolczak K., Konieczna L., Kostka T. (2018). Testosterone and dihydrotestosterone reduce platelet activation and reactivity in older men and women. *Aging*.

[60] Machlus K. R., Italiano J. E. (2013). The incredible journey: from megakaryocyte development to platelet formation. *The Journal of Cell Biology*.

[61] Eto K., Kunishima S. (2016). Linkage between the mechanisms of thrombocytopenia and thrombopoiesis. *Blood*.

